# The Prevalence of MRSA Nasal Carriage in Preoperative Pediatric Orthopaedic Patients

**DOI:** 10.1155/2016/5646529

**Published:** 2016-09-05

**Authors:** J. J. Walrath, W. L. Hennrikus, C. Zalonis, A. M. Dyer, J. E. Latorre

**Affiliations:** ^1^Department of Emergency Medicine, Yale-New Haven Hospital, New Haven, CT, USA; ^2^Department of Orthopaedics, Penn State Milton S. Hershey Medical Center, Hershey, PA, USA; ^3^Department of Infectious Diseases, Penn State Milton S. Hershey Medical Center, Hershey, PA, USA; ^4^Department of Public Health Sciences, Penn State College of Medicine, Hershey, PA, USA; ^5^Penn State College of Medicine, Hershey, PA, USA

## Abstract

Nasal carriage of methicillin-resistant* Staphylococcus aureus* (MRSA) has been described as a risk factor for postsurgical infection. The purpose of this study is to determine the prevalence of MRSA in pediatric orthopaedic patients and whether being a MRSA carrier is a predictor of postoperative infection. Six hundred and ninety-nine consecutive pediatric patients who underwent MRSA nasal screening prior to surgery were studied. Postoperative cultures, total surgical site infections (SSIs), and epidemiological and surgical prophylaxis data were reviewed. Forty-four of 699 patients (6.29%) screened positive for MRSA. Nine of the 44 patients (20.5%) that screened positive for MRSA had a subsequent SSI compared to 10 of the 655 patients (1.52%) that screened negative (*p* < 0.05). All 9 patients with a SSI had myelomeningocele. The prevalence of MRSA was 6.30% and was predictive of postoperative infection. Children with myelomeningocele were at the highest risk for having a positive MRSA screening and developing SSI.

## 1. Introduction

Methicillin-resistant* Staphylococcus aureus* (MRSA) is bacterial pathogen responsible for a variety of infections in both children and adults [1–4]. Since its first isolation by Jevons in 1960, MRSA has become progressively more widespread in hospitals and the community, as well as being increasingly difficult to treat [5, 6]. The prevalence of MRSA colonization has been found to be 0.18%–7.2% [7–9] in the general inpatient setting and up to 4%–8% in intensive care units [10]. A Welsh study reported colonization rates to be as high as 5.3% in orthopaedic and surgical wards [6]. Previous authors have suggested that the risk for subsequent surgical site infections (SSIs) with MRSA in asymptomatic, preoperative nasal carriage of MRSA increases [11–14]. MRSA SSIs are of particular concern, as these infections are associated with substantial morbidity and mortality, longer hospital stays, higher rates of rehospitalization, and increased cost of health care [15–20].

Risk factors for MRSA colonization have been well described [21–23]. Acquisition of this organism is typically associated with particular settings such as health care institutions like hospitals and long-term care facilities [24–26]. Then there are certain patient groups like those with prolonged hospitalization, past antimicrobial use, indwelling catheters, decubitus ulcers, use of intravenous drugs, treatment with enteral feedings or dialysis, and postoperative surgical wounds [25–27]. SSI can be caused by endogenous transmission of* Staphylococcus aureus* from the nose and skin to the surgical site [28].

Many studies have looked into the prevalence of MRSA in adult patients; however, few studies have looked at pediatric orthopaedic surgical patients [29]. The purpose of this study is to determine the prevalence of MRSA nasal carriage in preoperative pediatric orthopaedic patients, to identify risk factors of SSI due to MRSA, and to determine if MRSA nasal carriage is a predictor of postoperative infection in these patients. We hypothesized that the prevalence rate and risk factors in pediatric patients would be similar to those in adults. We also propose that MRSA nasal carriage would be predictive of postoperative infection in our pediatric population.

## 2. Materials and Methods

The study protocol was reviewed and approved by the Penn State College of Medicine Institutional Review Board (IRB). 699 consecutive patients under the age of 18 years, who underwent orthopaedic or spinal neurosurgery between April 2009 and March 2011, were retrospectively studied from the MRSA screening registry maintained by the Department of Infectious Disease at our institution.

As part of an ongoing hospital surveillance program, swabs of the anterior nares are performed on all patients admitted who do not have a known prior diagnosis of MRSA, except for those patients in the obstetrical wing. Nasal swabs are collected within 48 hours of admission and processed using the BD GeneOhm™ MRSA Assay (Becton Dickinson Diagnostics, San Diego, CA). This method allows for direct detection of MRSA from the nasal specimen. It has manufacturer-reported sensitivity of 93% and specificity of 96%. This PCR-based assay detects unique gene sequences for identification of both* S. aureus* (orfX) and methicillin resistance (SCCmec) [30, 31]. During the time of this study, the surgeon was not made aware of the MRSA screening result until after the surgery is performed. Therefore, no special interventions were performed preoperatively in patients with positive MRSA nasal screening.

Patient charts were reviewed for demographic information (age and gender), diagnosis, tobacco use, comorbidities (diabetes, immunodeficiency, and renal deficiency), corticosteroid use, location of residence prior to hospitalization, prior hospitalization, type of surgery, skin preparation, antibiotic prophylaxis, class of wound contamination, and surgical site infection as defined by NSQIP as an infection occurring within 30 days of an operation at the site of the procedure at the superficial, deep, or organ level [32]. The primary aim was to identify risk factors for MRSA colonization, SSI (as defined by NSQIP as an infection within 30 days of an operation), and SSI due to methicillin-resistant* Staphylococcus aureus *by using univariate analysis of various factors including gender, diagnosis, prior hospitalization, underlying disorder, tobacco use/exposure, and corticosteroid use. The secondary aims were (i) to determine the prevalence of preoperative nasal carriage of MRSA in our cohort of patients and (ii) to estimate the risk of* S. aureus* SSI.

Fisher's exact test was used for the analysis of categorical variables. The Wilcoxon nonparametric test was used for mean comparisons. *p* values below 0.05 were considered to be statistically significant.

## 3. Results

A total of 699 patient charts were reviewed. The baseline characteristics of the patients are shown in Table 1. 55% of the patients were males and 45% were females. The average age of the patients was 9.54 years with a range of 0 to 18 years. Forty-four patients (6.29%) screened positive for MRSA by nasal swab on admission. Twenty-eight of 44 patients (63.6%) that screened positive for MRSA were male.

Univariate analyses were performed to assess the risk factors for MRSA colonization, SSI overall, and SSI with MRSA (Table 2). The study identified prior hospitalization, the diagnoses of myelomeningocele, cerebral palsy and Chiari malformation, and other infections as risk factors for MRSA colonization. Risk factors for SSI overall included the diagnoses of myelomeningocele, Chiari malformation, and renal disease, as well as corticosteroid use prior to surgery. Risk factors for SSI with MRSA were diagnoses of myelomeningocele and renal disease.

Postoperatively, nine of 44 (20.5%) patients that screened positive for MRSA had a subsequent surgical site infection compared to 10 of 655 (1.52%) patients that screened negative for nasal MRSA (*p* value < 0.001). Eight patients that screened positive for MRSA and had a SSI with MRSA also had the diagnosis of myelomeningocele. Overall, 203 of 699 patients (29%) had myelomeningocele. Children with myelomeningocele were more likely to screen positive for MRSA. For example, 18 out of 203 (8.87%) patients with myelomeningocele screened positive for MRSA compared to only 5.9% who did not have myelomeningocele. Among myelomeningocele patients, those that screened positive for MRSA were more likely to develop a SSI than those who screened MRSA negative on admission (44.4% versus 2.2%, *p* value < 0.001). Eight out of 203 (3.9%) of myelomeningocele patients had a SSI with MRSA. Among patients without myelomeningocele, none developed a surgical site infection and no difference was detected between MRSA screening groups.

In addition, 88.9% of the MRSA positive and SSI positive patients were previously hospitalized at least once compared to 74.3% of the MRSA positive and SSI negative patients and 51.3% of the MRSA negative patients. Approximately 55% of patients in the study population had been previously hospitalized. 91% of these patients were admitted from their homes, 3.3% were newborns, 0.6% came from a group home, and 4.9% were transferred from an outside hospital.

No patient with a MRSA + nasal swab and a subsequent MRSA SSI had received vancomycin as a preoperative antibiotic. Antibiotic prophylaxis in this group of patients included cephazolin, ceftriaxone, or nafcillin. Three patients with a MRSA + nasal swab and no subsequent SSI had received vancomycin as a preoperative antibiotic. Antibiotic prophylaxis in this group of patients also included ampicillin, cefazolin, clindamycin, gentamycin, nafcillin, and zosyn.

Skin preparation in the patients who demonstrated a MRSA + nasal swab and a subsequent MRSA SSI included bacitracin and betadine. Skin preparation in the patients who demonstrated a MRSA + nasal swab and no subsequent SSI included bacitracin, bacitracin polymyxin B, betadine, chlorhexidine, and DuraPrep. SSI can decrease but cannot be prevented by adequate skin preparation [22].

All patients who were MRSA+ on admission and developed a surgical site infection were found to be growing MRSA bacteria. For those who had a SSI and were MRSA negative on admission, eight were positive for methicillin-sensitive* S. aureus* (MSSA), one for pseudomonas, and one for MRSA. Notably, the only patient who grew MRSA had multiple diagnoses of myelomeningocele, infection, and Chiari malformation.

## 4. Discussion

Decreasing SSI is a major focus in patient safety and quality programs. The increasing prevalence of community-acquired MRSA and current antibiotic-prescribing trends make MRSA a concern for the orthopaedic surgeon [33, 34]. No general consensus exists concerning the optimal preoperative decolonization and/or prophylaxis of patients who are colonized with MRSA [35–41]. However, identifying potential risk factors for MRSA acquisition in children may help prevent subsequent SSIs.

There are several notable findings reported in the current study. The first finding is that the prevalence of nosocomial carriage of MRSA in our pediatric population was 6.29%. This pediatric prevalence rate is consistent with those found in adult populations on orthopaedic and surgical wards [6]. The second finding is that pediatric patients who were MRSA positive on the nasal screening before surgery were significantly more likely than patients who were MRSA negative on the preoperative nasal screening to have a postoperative SSI. All patients who were MRSA positive and developed a SSI were found to be growing MRSA and not another bacterium. This suggests a high likelihood of developing a SSI secondary to MRSA if one is MRSA positive on admission and supports the fact that nasal carriage of MRSA at the time of surgery is a risk factor for MRSA SSI [34–40]. In addition, patients who were MRSA negative and obtained a SSI were predominantly due to MSSA and not MRSA. This signifies that emphasis in screening for colonization of both MRSA and MSSA presurgically may be of benefit.

Furthermore, children with a diagnosis of renal failure or infection prior to surgery who were MRSA positive on admission were significantly more likely to develop a SSI than their MRSA negative counterparts. This has the potential of being important knowledge prior to pediatric surgeries to ensure that special measures can be taken with known MRSA carriers. Children with myelomeningocele were at the highest risk of being positive for MRSA on nasal PCR testing and for postoperative SSI with MRSA. This supports prior studies suggesting that patients with myelomeningocele are at the highest risk for SSI after posterior spinal fusion [42, 43]. In general, myelomeningocele has a higher risk of hospitalization, antibiotic use, and surgical exposure.

This study has a few limitations. For example, variations among populations in* S. aureus* carriage and infection by geographic location raise questions about the generalizability of these data from central Pennsylvania to other populations. Moreover, sampling was limited to the anterior nares. Although this site is considered the primary colonization site for MRSA, other sites including the throat, groin, axilla, and perianal areas can be colonized. Therefore, using a single nasal swab, the carriage frequency of MRSA in our cohort may be underestimated. The preoperative skin preparations were variable and not controlled. In addition, our institution uses the BD GeneOhm™ MRSA Assay as the standard screening protocol. No other enrichment media are typically used. This means that our sensitivity in ruling out MRSA was not as high as it could have been.

## 5. Conclusions 

In conclusion, the optimization of perioperative methods to prevent infection is essential. In the current study, myelomeningocele, renal disease, and a current infection were significant risk factors on postoperative infection in MRSA positive patients. Future studies should target myelomeningocele patients for infection control and treatment as well as those who have an infection at the time of surgery and patients with renal failure.

## Figures and Tables

**Table 1:** Characteristics of the 699 patients included in the study with special emphasis on patients who tested MRSA positive on admission.

	MRSA+		MRSA−		Total
	SSI+, *n* (%)	SSI−, *n* (%)	SSI+, *n* (%)	SSI−, *n* (%)	*n* (%)
	(*n* = 9)	(*n* = 35)	(*n* = 10)	(*n* = 645)	(*n* = 699)
Age, mean (range)	5.55 (0–18)	8.14 (0–17)	6.67 (0–15)	8.91 (0–18)	9.54 (0–18)
Gender					
Male	7 (77.8)	21 (60.0)	6 (60.0)	352 (54.6)	386 (55.2)
Female	2 (22.2)	14 (40.0)	4 (40.0)	293 (45.4)	313 (44.8)
Location prior to hospitalization					
Home	7 (77.28)	29 (82.9)	9 (90.0)	593 (91.9)	638 (91.3)
Newborn	2 (22.2)	3 (8.57)	0 (0.0)	18 (2.8)	23 (3.3)
Group home	0 (0.0)	1 (2.85)	0 (0.0)	3 (0.5)	4 (0.6)
Other hospital	0 (0.0)	2 (5.71)	1 (10.0)	31 (4.8)	34 (4.9)
Prior hospitalization in last 5 years					
Yes	8 (88.9)	26 (74.3)	5 (50.0)	331 (51.3)	370 (52.9)
No	1 (11.1)	9 (25.7)	5 (50.0)	314 (48.7)	329 (47.1)
Diagnosis^*∗*^					
Myelomeningocele	8 (88.9)	10 (28.6)	4 (40.0)	181 (28.1)	203 (29.0)
Cerebral palsy	0 (0.0)	3 (8.6)	0 (0.0)	34 (5.3)	37 (5.3)
Trauma	0 (0.0)	6 (17.1)	1 (10.0)	149 (23.1)	156 (22.3)
Tumor	0 (0.0)	2 (5.7)	1 (10.0)	52 (8.1)	55 (7.9)
Infection	1 (11.1)	7 (20.0)	1 (10.0)	34 (5.3)	43 (6.2)
Scoliosis	0 (0.0)	2 (5.71)	0 (0.0)	95 (14.7)	97 (13.9)
Chiari malformation	2 (22.2)	1 (2.85)	2 (20.0)	33 (5.1)	38 (5.4)
Other	1 (11.1)	9 (25.7)	3 (30.0)	142 (22.0)	154 (22.0)
Surgery					
Neurosurgery	9 (100)	20 (57.1)	9 (90.0)	359 (55.7)	397 (56.8)
Orthopaedic surgery	0 (0.0)	15 (42.9)	1 (10.0)	286 (44.3)	302 (43.2)
Wound class					
Clean	9 (100.0)	24 (68.6)	10 (100.0)	590 (91.5)	633 (90.6)
Clean-contaminated	0 (0.0)	8 (22.9)	0 (0.0)	38 (5.9)	46 (6.6)
Contaminated	0 (0.0)	3 (8.6)	0 (0.0)	11 (1.7)	14 (2.0)
Infected	0 (0.0)	0 (0.0)	0 (0.0)	6 (0.9)	6 (0.9)
Underlying disorder					
Diabetes mellitus	0 (0.0)	0 (0.0)	0 (0.0)	6 (0.9)	6 (0.9)
Immunocompromised	1 (11.1)	2 (5.7)	1 (10.0)	24 (3.7)	28 (4.0)
Renal disease	4 (44.4)	0 (0.0)	1 (10.0)	39 (6.0)	44 (6.8)
Tobacco use/exposure					
Yes	0 (0.0)	1 (2.9)	0 (0.0)	34 (5.3)	35 (5.0)
No	9 (100.0)	34 (97.1)	10 (100.0)	611 (94.7)	664 (95.0)
Corticosteroid use (%)					
Yes	3 (33.3)	6 (17.1)	0 (0.0)	52 (8.1)	61 (8.7)
No	6 (76.7)	29 (82.9)	10 (100.0)	593 (91.9)	638 (91.3)

^*∗*^Many individuals had multiple diagnoses and were thus counted twice.

**Table 2:** Univariate analysis of risk factors for MRSA colonization, SSI overall, and SSI due to MRSA.

Risk factors	MRSA colonization	SSI overall	SSI due to MRSA
	*p* value	*p* value	*p* value
Male gender versus female gender	0.2	1	0.69
Prior hospitalization	0.001	0.10	0.41
Diagnosis			
Myelomeningocele	<0.001	<0.001	<0.001
Cerebral palsy	0.035	1	1
Trauma	0.33	0.15	0.32
Tumor	1	1	1
Infection	<0.001	0.21	1
Scoliosis	0.21	0.15	1
Chiari malformation	0.043	<0.001	0.092
Other	0.058	0.60	0.32
Underlying disorder			
Diabetes mellitus	1	1	1
Immunocompromised	0.40	0.17	0.49^a^
Renal disease	0.51	0.0098	0.003
Tobacco use/exposure	0.16	0.41	1
Corticosteroid use	0.088	0.001	0.61

^a^One-tail test.
